# Inflammatory pseudo-tumor of the liver: a rare pathological entity

**DOI:** 10.1186/1477-7819-9-5

**Published:** 2011-01-23

**Authors:** Walid Faraj, Hana Ajouz, Deborah Mukherji, Gerald Kealy, Ali Shamseddine, Mohamed Khalife

**Affiliations:** 1 Department of Surgery, HPB and liver transplantation unit, American University of Beirut Medical Center, Beirut, Lebanon

## Abstract

Inflammatory pseudo-tumor (IPT) of the liver is a rare benign neoplasm and is often mistaken as a malignant entity. Few cases have been reported in the literature and the precise etiology of inflammatory pseudotumor remains unknown. Patients usually present with fever, abdominal pain and jaundice. The proliferation of spindled myofibroblast cells mixed with variable amounts of reactive inflammatory cells is characteristics of IPT. We reviewed the literature regarding possible etiology for IPT with a possible suggested etiology.

## Introduction

Inflammatory pseudo-tumor (IPT) of the liver is a rare benign neoplasm and is often mistaken as a malignant entity. They were first described in the lung in 1939 and have been reported subsequently in numerous locations, including the liver, spleen, lymph nodes, spinal cord, salivary glands, breast, and soft tissues. Liver involvement was first described in 1953 by Pack and Baker and may lead to biliary obstruction, portal hypertension, cirrhosis, and eventually hepatic failure. Few cases have been reported in the literature with no recognized etiology for IPT [1-4]. Patients usually present with fever, abdominal pain and jaundice. The proliferation of spindled myofibroblast cells mixed with variable amounts of reactive inflammatory cells is characteristic of IPT. IPT is sometimes misdiagnosed as a malignant tumor based on radiographic findings. We reviewed the literature regarding possible etiology for IPT with a possible suggested etiology.

### Exaggerated Inflammatory response

Inflammatory pseudotumour (IPT) of the liver, also known as inflammatory myofibroblastic tumor is a rare benign condition, which has been diagnosed with increasing frequency because of recent advances in imaging techniques. It is associated with many disease entities including Crohn's disease [5], diabetes mellitus [6], Sjögren's syndrome [7], gout [8], chronic ascending cholangitis, primary sclerosing cholangitis [9], Kostmann's disease [10], acute myeloblastic leukemia [11], HIV [12,13] and autoimmune pancreatitis [14,15]. Possible etiologies that would cause such an exaggerated inflammatory response include the possibilities of an unidentified infectious agent, autoimmune phenomena and systemic inflammatory response syndrome.

Several mechanistic etiologies have been proposed which includes increased biliary concentration of monomeric bile acids. This biochemical aberration can lead to bile duct injury and pericholangitis followed by onion skin type periductal fibrosis and finally chronic sclerosing cholangitis. This was mechanism has been elucidated and strengthened by three different and separate experimental models: 1) feeding lithocholic acid (LCA) (a monomeric bile acid) to mice [16] 2) common bile duct ligation which consequently lead to increased monomeric bile acid in bile ducts [17,18] and3) Mdr2/Mrd3 gene (gene responsible for canalicular phospholipid lipase) knock out mice [19] which results in an increased biliary concentration of monomeric bile acids in the absence of biliary phospholipid secretion and its equivalent in humans. All three of these experimental mouse models will result in inflammation of the biliary ducts of the mouse.

The assumed pathophysiology resulting from lithocholic acid toxicity in the liver was described by Fickert et al. He suggested that lithocholic acid leads to alterations of tight junctions of biliary epithelial cells resulting in leaky bile ducts and chemotaxis of neutrophil granulocytes. This is followed by inflammatory reaction and extravasation of toxic bile which lead to subepithelial fluid accumulation, and concomitant detachment of the biliary epithelium resulting in lifting of the epithelial cell layer. After which, neutrophil granulocytes invade the bile duct lamina via transmigration and ulcerations of the epithelial cell layer. Then the ongoing efflux of toxic bile into the portal field; together with activation of biliary epithelial cells, leads to proliferation and activation of periductal myofibroblasts resulting in rapidly evolving periductal fibrosis.

### IPT relationship with other pathologies

#### IPT and sclerosing cholangitis

Patients with thickened bile ducts and stricture formation and dilation of the duct distal proximal to that point. Many of the cases were not initially diagnosed as having sclerosing cholangitis [9].

#### IPT and Crohn's disease

The pathology showed stricture in the common bile duct (CBD) and dilated intrahepatic ducts. There is some evidence of biliary stricture disease consistent with and similar to primary sclerosing cholangitis. In addition, some patients have atypical biliary duct epithelial cells which could be caused by intraductal LCA crystals that damage BECs (biliary endothelial cells) as the result of the hydrophobic and lithogenic physicochemical properties of LCA [5,16] (knowing that LCA crystals could only be seen by electron microscopy).

#### IPT and autoimmune pancreatitis

Swelling of the pancreatic head could have lead to disruption in the flow of bile and therefore increase concentration of monomeric acid, which lead to bile duct hyperplasia, and formation of IPT. The pathology was reversed with steroids [15,16].

#### IPT and gastrointestinal stromal tumor (GIST) of the ileum

The presence of an ileal GIST could potentially disrupt bile acid absorption and the obstruction in the tumor could cause increased concentration of bile acid. Similar pathologic changes occur in-patient with ulcerative colitis in which large amounts of lithocholic acid is absorbed and presented to a susceptible liver [17,18,20].

#### IPT with recurrent pyogenic cholangitis

Hanada et al correlated the finding of hypoattenuating areas of IPT on CT scans with chronic inflammatory infiltrates with foamy histiocytes, plasma cells and lymphocytes on pathology. Iso-attenuating and hyper-attenuating areas were correlated with fibroblastic proliferation. In all the reported cases, intrahepatic ducts were irregularly dilated and diffusely thickened. In addition, periductal white fibrosis was noted and the hepatic ducts contained dark brown to black muddy fragile stones in all such patients. The question was whether IPT could be caused by bile stasis, while recurrent inflammation and calculi provoke degeneration and necrosis of the bile duct wall with subsequent periductal abscess or formation of xanthogranulomas (as in the case of xanthogranulomatous cholecystitis). An important conclusion of these studies was that IPT could develop as one of the spectra of recurrent pyogenic cholangitis [21-25].

### Tight junction alterations of the biliary duct epithelium

Sakai et al [26] reported that IPT was shown to enhance by extravasation of contrast material in fibrous tissue on delayed-phase CT (3 to 6 minutes after injection of contrast material) where as no characteristic enhancement pattern is seen on the early phase (40 to 100 seconds). Early enhancement of CT scans has been reported to be related to vascularity, whereas delayed uptake may be caused by slow diffusion into the abnormally large extracellular space of the hepatic mass. This delayed enhancement suggests that there is disruption in the tight junctions of the biliary duct epithelium in IPT which might also be explained by LCA toxicity that leads to tight junction alterations preceding lifting and ulceration of the epithelial cell layer in the bile duct. Tight junction alterations may be of primary importance in LCA-induced cholangiopathy [17]. Tight junctions between hepatocytes also showed alterations of the ZO-1 pattern with distortion and widening indicative of dilated canaliculi resulting in leaky bile ducts and chemotaxis of neutrophil granulocytes and extravasation of toxic bile. In cases where obstruction or raised pressure in the biliary system is present, these lesions might result from rupture of the canals of Hering [27] with exposure of hepatocytes to bile acid concentrations in the millimolar range derived from bile leaking into the liver parenchyma [28].

### Idiopathic inflammatory strictures of extrahepatic biliary tree

Idiopathic benign inflammatory strictures of extrahepatic bile ducts and IPT may share a common etiology. Several patients with IPT have developed eosinophilia, which may serve to detoxify LCA in the periductal tissues. Kafeel et al [29] reported that IPT was regarded as an abnormal exuberant tissue response to some external stimulus [30] or pathology similar to retroperitoneal fibrosis [31]. While the mechanism leading to this condition remains unclear, extravasation of bile into the gallbladder wall, with involvement of Rokitansky-Aschoff sinuses, or extravasation through a small ulceration in the mucosa, appears to be a precipitating factor.

### Inborn errors of bile acid metabolism

In the rare cases of inborn errors of bile acid metabolism one of the prominent clinical features is liver and spleen enlargement, and the progression of the liver disease is most rapid when the defect results in accumulation of bile acids [32-34]. The liver disease may be transient, delayed in onset and mild. Pathologic findings in this disease include intralobular cholestasis with giant cell transformation, prevalence of necrotic hepatocytes including giant cell forms, and hepatic injury confined to the portal limiting plate where the smallest bile ductules may be injured, resulting in neocholangiolar proliferation. Giant cell transformation was thought to be the result of fusion of hepatocytes whenever toxic bile acids are present, similar pathology is seen in IPT.

### Elevated bile acid concentration

Increased bile acid concentration has been observed in many other entities. One of which is MDR3 deficiency, which is thought to lead to decreased excretion of cytoprotective biliary phospholipids, leaving an increased pool of cytotoxic biliary bile salts that gives rise to subsequent bile duct damage and proliferation and release of gamma glutamyl transpeptidase (GGTP) into the serum [35-40]. This was also noted in biliary atresia resulting in inflammatory and fibrous cells surrounding miniscule ducts, bile duct proliferation, severe cholestasis with plugging, and inflammatory cell infiltration [41].

Some authors suggested that all bile acids, at concentrations >25 μmol/L; induce a 2.5- to 3-fold increase in hepatic stellate cell proliferation via activation of the epidermal growth factor receptor. Bile acid-induced proliferation is mediated by activation of a protein kinase C/extracellular signal-regulated kinase/p70^S6K^-dependent pathway [42]. Bile acid does not only affect the liver, but exposure of airway epithelium to bile acids may induce a fibrotic response. Aspiration of bile acids may induce airway fibrosis through the production of TGF-β_1 _and fibroblast proliferation [43].

### Lithocholic acid and ITP

Lithocholic acid is a hydrophobic secondary bile acid that is primarily formed in the intestine by the bacterial 7α-dehydroxylation of chenodeoxycholic acid. It is poorly water-soluble and rather toxic to cells. In humans the harmful effects of LCA and other bile acids are attenuated by two hepatic detoxification pathways, namely hydroxylation and conjugation. These reactions make the bile acid more hydrophilic and facilitate its excretion in the feces or urine. It was interesting to find that lithocholic acid was present in the serum of patients with jaundice and, in smaller amounts, healthy adults [44]. In cholestasis, LCA levels increase in the liver and intestine [45]. The finding of lithocholic acid in blood is of interest because of its possible role in injuring human liver [44].

Recent studies suggest that LCA induces its own detoxification by activating nuclear receptors to promote transcription of genes encoding sulfotransferase. Lithocholic acid is a rare example of a toxic endobiotic; a variety of mechanisms work to detoxify it [46]. One involves the pregnane X receptor (PXR), a NR that controls hepatic detoxification pathways for harmful bile acids and several drugs (such as rifampicin and phenobarbitol) which are equivalent in humans to the steroid and xenobiotic receptor (SXR) or pregnane-activated receptor. Once activated by certain toxic secondary bile acids and other ligands, PXR attenuates bile acid production by directly inhibiting CYP7A1 (cholesterol 7α-hydroxylase, which catalyzes the rate-limiting step in the conversion of cholesterol to bile acids in the liver [47]). Through this receptor, certain steroids exhibit a protective effect against various types of intoxication. These "catatoxic" steroids afford protection against harmful chemicals by accelerating their metabolism. This might explain the cases of ITP were the pathology was reversed by steroid.

Pregnenolone and other catatoxic compounds stimulate the transcription of the CYP3A subfamily of cytochrome P450 monooxygenases, where they metabolize a wide variety of xenobiotics and natural compounds including steroids and bile acids. In 1970, Selye [48] showed that PCN (pregnenolone 16α-carbonitrile) prevented the LCA-induced hepatoxicity and mortality in rodents. PXR plays a fundamental role in protecting the body from toxic bile acids. PXR is activated by LCA and its 3-keto metabolite and coordinately regulates genes involved in the biosynthesis, transport, and metabolism of LCA. PXR protects the liver against pathophysiological levels of LCA [49].

Conjugated and unconjugated bile acids rapidly induce EGR and FOS gene expression as well as cytoplasmic mitogen-activated protein kinase (MAPK) activation. Of the bile acids, lithocholic acid appeared to be more potent than the other equimolar bile acid concentrations. The more hydrophobic bile acid, lithocholic acid, may induce the inflammatory cascade at relatively low concentrations because of its membrane-diffusing properties [50]. This mitogenic effect has some cell-specificity because treatment of unrelated 3T3 fibroblasts with high concentrations of lithocholic acid caused no detectable MAPK-induced MBP phosphorylation. The myofibroblastic proliferation in ITP might be from a transformed stellate cell. In response to stress, hepatic stellate cells undergo "activation" which connotes a transition from quiescent vitamin A-rich cells into proliferative, fibrogenic, and contractile myofibroblast. The major phenotypic changes after activation include proliferation, contractility, fibrogenesis, matrix degradation, chemotaxis, retinoid loss, and WBC chemoattraction [51].

In 1960s the effect of bile acid on raising human temperature was discussed in the Journal of Clinical Investigation. Palmer et al found out that LCA is the most potent of the naturally occurring steroids that produce intense fever and inflammation in man. The endogenous biliary steroid, lithocholic acid, has significant inflammatory and pyrogenic action in man [51]. It was interesting to find that six milligrams of lithocholate injected intramuscularly or intravenously is sufficient to produce intense fever and local inflammation in humans which might explain the high grade fever which present in patients with IPT.

### The immunohistochemistry (IHC) of IPT

The smooth muscle actin (SMA) and vimentin are usually positive in stellate and spindle cells, whereas desmin, CD34, S100 protein, and anaplastic lymphoma kinase (ALK) are negative [52,53].

It is likely that many spindle cells correspond to activated histiocytes as they coexpress vimentin and macrophage-associated markers; they are intermingled with vimentin-positive fibroblasts and variable numbers of vimentin and actin positive myofibroblasts [54]. Because of the variable immunophenotypic patterns seen in hepatic IPT, it is possible that they arise from a common mesenchymal cell that is capable of differentiating along different pathways. The majority would develop a myofibroblastic phenotype and be positive for SMA and vimentin [55]. Many SMA-positive myofibroblastic cells were found in IPT, hence suggesting an ongoing fibrous process.

Inflammatory pseudotumor of the liver constitutes a diagnostic and therapeutic challenge. In the presence of a solitary liver lesion, with clinical and laboratory features suggesting active inflammation, the diagnosis of inflammatory pseudotumor should be considered. Proper investigation to exclude malignancy should be undertaken, resection of the lesion should be considered when in doubt.

Several theories have been proposed for the possible etiology of inflammatory pseudotumour of the liver. We think that the significant inflammatory effect of the biliary steroid lithocholic acid is a major contributor in the formation of those benign lesions in the liver.

## Competing interests

The authors declare that they have no competing interests.

## Authors' contributions

WF drafted the manuscript, HA and DM participated in the design of the study, GK and MK participated in the design and coordination of the study. All authors read and approved the final manuscript.
